# Editorial: New insights into multiple endocrine neoplasia type 1

**DOI:** 10.3389/fendo.2023.1266148

**Published:** 2023-08-11

**Authors:** Anne Barlier, Pauline Romanet, Natalia S. Pellegata

**Affiliations:** ^1^Aix Marseille Univ, APHM, INSERM, MMG, Laboratory of Molecular Biology Hospital La Conception, MarMaRa Institute, Marseille, France; ^2^Institute for Diabetes and Cancer, Helmholtz Zentrum München, Neuherberg, Germany; ^3^Joint Heidelberg-IDC Translational Diabetes Program, Heidelberg University Hospital, Heidelberg, Germany; ^4^Department of Biology and Biotechnology “L. Spallanzani”, University of Pavia, Pavia, Italy

**Keywords:** MEN1 = multiple endocrine neoplasia Type 1, PitNET, pancreatic NETs, genetics, MEN1 non endocrine lesions

Multiple endocrine neoplasia type 1 (MEN1) is a rare hereditary disease described for the first time nearly 70 years ago by Paul Wermer. The genetic cause was identified only 43 years later. The name of the syndrome was used to name the causative gene: *MEN1*, encoding the menin protein. The MEN1 syndrome is characterized by a broad spectrum of clinical manifestations among which the three cardinal lesions are pituitary neuroendocrine tumors (PitNETs - mainly lactotroph, somatotroph or non-functional), primary hyperparathyroidism, and neuroendocrine duodeno-pancreatic tumors. At the clinical level, several issues remain unresolved.

Two articles of this MEN1-focused Research Topic focus on non-endocrine neoplastic manifestations, less explored in the literature, and address several issues: (i) what is the link between the *MEN1* genetic alteration and the lesion, what are the underlying molecular mechanisms? (ii) should these lesions be considered as MEN1-associated lesions and as such be taken into account as additional feature for the diagnosis of MEN1 disease? (iii) which is the frequency of these non-endocrine lesions in MEN1 patients? Consequently, should these lesions be monitored as breast cancer or non-pituitary cerebral tumor are? These questions are addressed in the beautiful review from Waguespack Frontiers | Beyond the “3 Ps”: A critical appraisal of the non-endocrine manifestations of multiple endocrine neoplasia type 1 (frontiersin.org) and in the study from Pierotti et al. (Frontiers | Cutaneous lesions and other non-endocrine manifestations of Multiple Endocrine Neoplasia type 1 syndrome (frontiersin.org)) Due to the fact that these non-endocrine MEN1 manifestations are not systematically investigated nor reported, data are still missing to answer with accuracy the questions illustrated above. However, these two papers significantly contribute to assessing the relevance of non-endocrine manifestations of MEN1 disease.

The remaining papers in this MEN1 Research Topic focus on PitNETs and neuroendocrine duodeno pancreatic tumors (DP-NET), lesions strongly impacting on the patients’ quality of life through significant short- and long-term morbidity. The first article by Miranda et al., (Frontiers | Clinical and molecular features of four Brazilian families with multiple endocrine neoplasia type 1 (frontiersin.org) reports 4 large and well characterized Brazilian MEN1 families, including 11 affected individuals. It is interesting to note that these 4 families were extracted by the authors from a large cohort of 500 patients, recorded in the University Hospital of Brasilia, a center for Pit-NETs. Among the affected individuals, 4 patients had a family history compatible with MEN1 and 30 with Familial Isolated Pituitary Adenomas (FIPA). This work emphasizes the importance to sequence the *MEN1* gene in young patients presenting with PitNETs, which could represent the first manifestation of the disease as previously reported (1). All MEN1 patients belonging to these 4 families had primary hyperparathyroidism (PHPT), sometimes completely asymptomatic, supporting previous findings stating that the penetrance of HPT is around 100% in MEN1 (2).

DP-NETs rank second among the MEN1-associated lesions in terms of frequency, and represent the main cause of death for the patients. We agree with van Beek et al. Frontiers | Diagnosing pancreatic neuroendocrine tumors in patients with multiple endocrine neoplasia type 1 in daily practice (frontiersin.org) that considering the management of MEN1-related DP-NETs is crucial in the clinical management of MEN1 disease. Consequently, in this study conducted on a large cohort of 413 patients who underwent 3477 imaging scans, the authors estimated the accuracy and specificity of MRI vs. CT for the screening and follow up of DP-NETs and concluded that MRI should be the preferred modality as it is non-invasive and also has the advantage of being associated to lower radiation exposure.

In 2023, the screening program for MEN1 patients leads to discovery of small pancreatic-NETs (P-NET), often asymptomatic in case of non-functional lesion. This put the clinician in a complex situation, having to consider: (i) the risk of pancreatic surgery causing high morbidity; (ii) the risk of metastasis from MEN1-related P-NETs; (iii) the risk of high recurrences of P-NETs in the remaining pancreatic gland. Therefore, P-NETs management remains highly controversial. In the paper from van Vliembergen et al., Frontiers | Precision radiotherapy using MR-linac for pancreatic neuroendocrine tumors in MEN1 patients (PRIME): a protocol for a phase I-II trial, and systematic review on available evidence for radiotherapy of pNETs (frontiersin.org), the authors report an exhaustive review of the literature on the radiotherapy of MEN1-related P-NETs. The main point of this review is that these lesions can be considered radiosensitive, although with the drawback of major complications secondary to radiotherapy. Indeed, the fact that the location of the pancreas depends on posture and breathing, together with several other technical difficulties, have limited the use of radiotherapy in clinical practice due to local complications. Considering the radiosensitivity of P-NETs, the authors set up a challenging clinical trial using magnetic resonance-guided radiation therapy delivered by MR-Linac, a combination of linear accelerator and an MRI scanner, to specifically target even small P-NETs. This approach could open new horizons for patients with these tumors.

In conclusion, MEN1 is perhaps an “old” syndrome, but numerous challenges still remain in the screening program, diagnosis and in the lesion management specifically for P-NETs, aimed at preserving the quality of life of these patients finally bearing “ a chronic disease.”

## Author contributions

AB: Writing – original draft. PR: Writing – review & editing. NP: Writing – review & editing.
